# Inhibitory effects of ultrasound irradiation on *Staphylococcus epidermidis* biofilm

**DOI:** 10.1007/s10396-021-01120-3

**Published:** 2021-08-19

**Authors:** Harumi Koibuchi, Yasutomo Fujii, Yusuke Sato’o, Takashi Mochizuki, Toshiyuki Yamada, Longzhu Cui, Nobuyuki Taniguchi

**Affiliations:** 1 Department of Clinical Laboratory Medicine, Jichi Medical University, 3311-1 Yakushiji Shimotsuke-Shi, Tochigi, 329-0498 Japan; 2 Department of Human Health Science, Kyoto University Graduate School of Medicine, Kyoto, Japan; 3 Division of Bacteriology, Department of Infection and Immunity, Jichi Medical University, Tochigi, Japan; 4 Medical Ultrasound Laboratory Co., Ltd., Tokyo, Japan

**Keywords:** Biofilm, *Staphylococcus epidermidis*, Ultrasound, Catheter-related bloodstream infection, Acoustic parameter

## Abstract

**Purpose:**

We aimed to investigate whether low-intensity continuous and pulsed wave ultrasound (US) irradiation can inhibit the formation of *Staphylococcus epidermidis* biofilms, for potential application in the treatment of catheter-related bloodstream infections (CRBSI).

**Methods:**

*S. epidermidis* biofilms that formed on the bottom surfaces of 6-well plates were irradiated on the bottom surface using the sound cell incubator system for different intervals of time.

**Results:**

US irradiation with continuous waves for 24 h notably inhibited biofilm formation (*p* < 0.01), but the same US irradiation for 12 h had no remarkable effect. Further, double US irradiation with pulsed waves for 20 min inhibited biofilm formation by 33.6%, nearly two-fold more than single US irradiation, which reduced it by 17.9%.

**Conclusion:**

US irradiation of a lower intensity (*I*_SATA_ = 6–29 mW/cm^2^) than used in a previous study and lower than recommended by the Food and Drug Administration shows potential for preventing CRBSI caused by bacterial biofilms.

## Introduction

The use of central venous catheterization has become important in the management of critical patients. Insertion of central venous catheters makes vascular access secure [1]. Catheter-related bloodstream infection (CRBSI) is a serious infectious disease in hospitalized patients because it increases the length of stay, cost of care, and risk of hospital death [2]. The cost of CRBSI ranges between $33,000 and $75,000, depending on the type of intensive care unit [3]. The most common causative agents of CRBSI are coagulase-negative *staphylococci* such as *Staphylococcus epidermidis* (*S. epidermidis*). This bacterium ubiquitously colonizes the skin and invades the bloodstream through skin-inserted medical devices, such as intravascular catheters and joint prostheses [4]. Biofilm formation is essential for the pathogenicity of *S. epidermidis* [5]. The biofilm matrix prevents the access of antibiotics to bacterial cells. Alternatively, the slow growth of bacteria in the mature biofilm protects the cells from antibiotics [6]. Therefore, biofilm-forming bacteria are exceptionally resistant to antibiotics, and biofilm-associated infection is one of the most serious problems in hospitalized patients.

Ultrasound (US) energy has a chemical and biological effect on cells [7]. Considerable attention should be paid to US intensity when US irradiation is used for therapeutic purposes in clinical settings because US energy has biological effects on normal cells. The Food and Drug Administration (FDA) has recommended guidelines for the output of diagnostic ultrasound medical devices. This guideline recommends an upper limit level of US intensity of 720 mW/cm^2^ (*I*_SPTA_) on peripheral vessels [8].

The effectiveness of US irradiation in biofilm eradication has been reported previously; however, the US intensity used in these studies is too high according to FDA guidelines for clinical therapy applications [9, 10]. In our previous study, US irradiation decreased the amount of *S. epidermidis* biofilm produced on the bottom surface of wells in a 6-well plate [11]. The biofilm was irradiated with 1-MHz continuous-wave US in this study. US intensity was *I*_SPTA_ = 1.66 mW/cm^2^, and the irradiation time was 24 h. This intensity was lower and the time was longer than those in other similar studies [9, 10].

Furthermore, previous studies focused on using US irradiation for the eradication of formed biofilms. However, if a biofilm is destroyed by US irradiation, bacteria present in and around the biofilm may disperse into the bloodstream and cause bacteremia in the patient. Therefore, for treating CRBSI, it is important to prevent bacteria from forming a biofilm rather than destroying a completely formed biofilm.

In this study, we used sound cell incubator (SCI) as a US irradiation system. The purpose of the study was to evaluate the inhibitory effect of US irradiation with a lower intensity level than that used in previous studies on biofilm formation.

## Materials and methods

### Preparation of bacterial solution

*S. epidermidis* (ATCC 35984 RP 62A) was purchased from American Type Culture Collection (ATCC, Virginia, USA) and stocked in Microbank (Iwaki, Tokyo, Japan). Bacterial cells were inoculated on Tryptic Soy Agar plates (Becton–Dickinson and Company, Sparks, USA) at 37 °C in 5% CO_2_ for 24 h. We inoculated five or six colonies of *S. epidermidis* in 5 mL of Brain Heart Infusion (BHI, Becton–Dickinson and Company) in 15-mL Corning tubes (Corning, Glendale, Arizona, USA). The tubes were incubated at 37 °C in 5% CO_2_ for 18 h.

### Biofilm grown in 6-well plates

Twenty μL of bacterial culture was added to 2 mL of BHI containing 1% D-glucose (Wako, Osaka, Japan) in 6-well plates (Thermo Fisher Scientific, NY, USA). After incubation, media were discarded and the wells were washed four times using physiological saline. White patches left on the bottom surfaces of the wells after washing were considered “biofilm.”

### Quantification of biofilms

The amount of biofilm was quantified by crystal violet staining. The biofilm was stained with 2 mL of 1% (w/v) crystal violet (Merck KGaA, Darmstadt, Germany) for 30 min. After incubation, the dye was discarded and the wells were gently washed twice with distilled water. To extract the stain, a mixture of 1% (v/v) hydrochloric acid and 70% (v/v) ethanol was added to the wells and the extracted solutions were diluted 20 times. The amount of biofilm was determined by measuring the absorbance of the diluted solution at 595 nm.

### US irradiation system

**Fig. 1:**
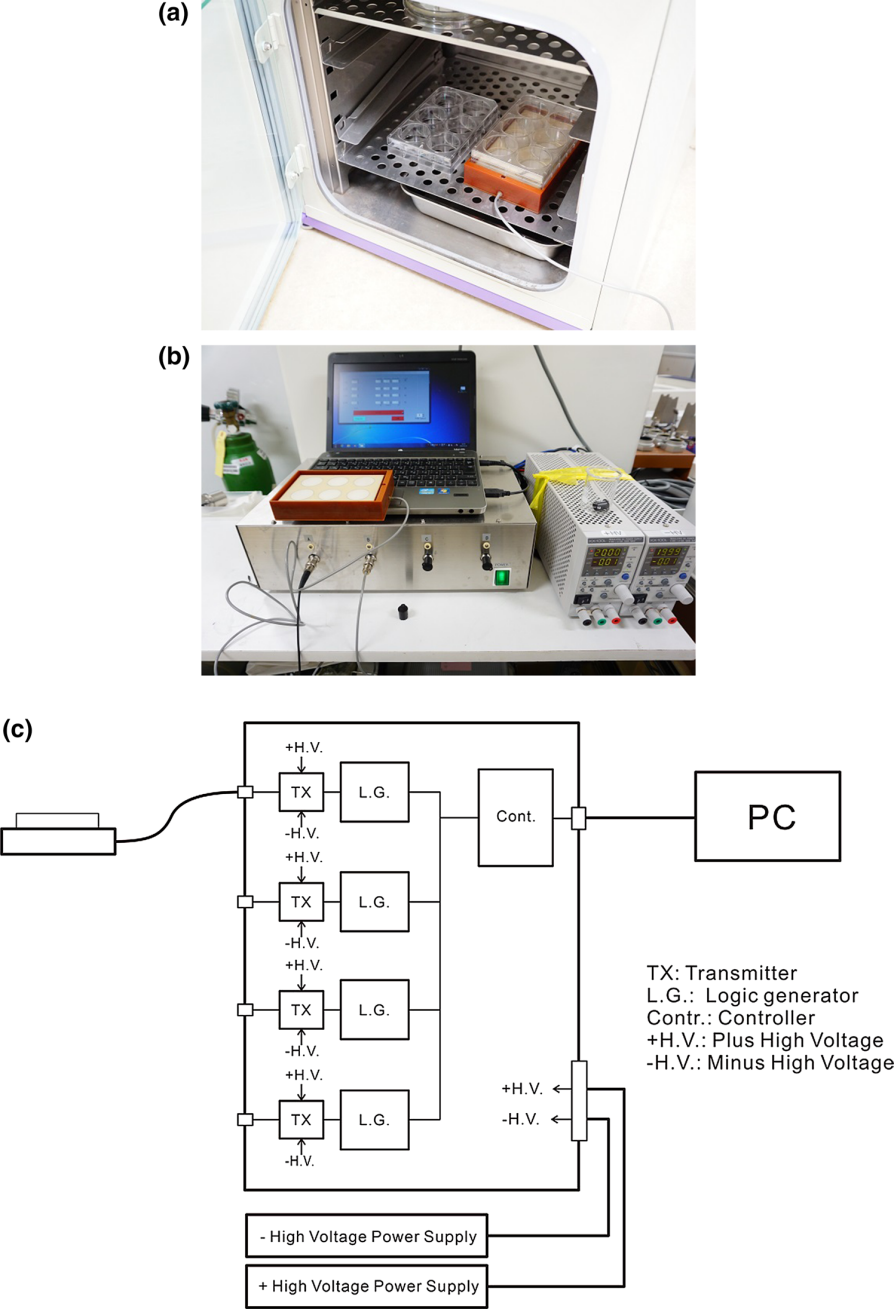
**a** US irradiation can be carried out in the cell incubator by SCI. **b** This system consists of driving unit SCI-D100, a personal computer with software to control it, plus and minus power supply units, and irradiator SCI-m1-6 (put on the PC in the photogram) for irradiation of a 6-well plate. **c** A schematic block diagram of the SCI system—which has four independent blocks of driving electric circuits, each of which consists of a logic generator and a pulse transmitter—is shown. Accordingly, one run of the experiment can be done under four different irradiation conditions at the same time

For ultrasound irradiation of cells in this study, the SCI system (Medical Ultrasound Laboratory, Tokyo, Japan) was employed, in which essential irradiation parameters such as ultrasound frequency, pulse repetition time (PRT), pulse duty ratio, duration of irradiation, and driving voltage can be easily and precisely set up as requested. The system also has a unique feature where the irradiation of cells shown in Fig. 1a could be carried out inside the cell incubator under low-intensity ultrasound and long duration of irradiation. The SCI system is shown in Fig. 1b. Figure 1c is a schematic block diagram of the SCI system.

### Measurement of US irradiation intensity

**Fig. 2:**
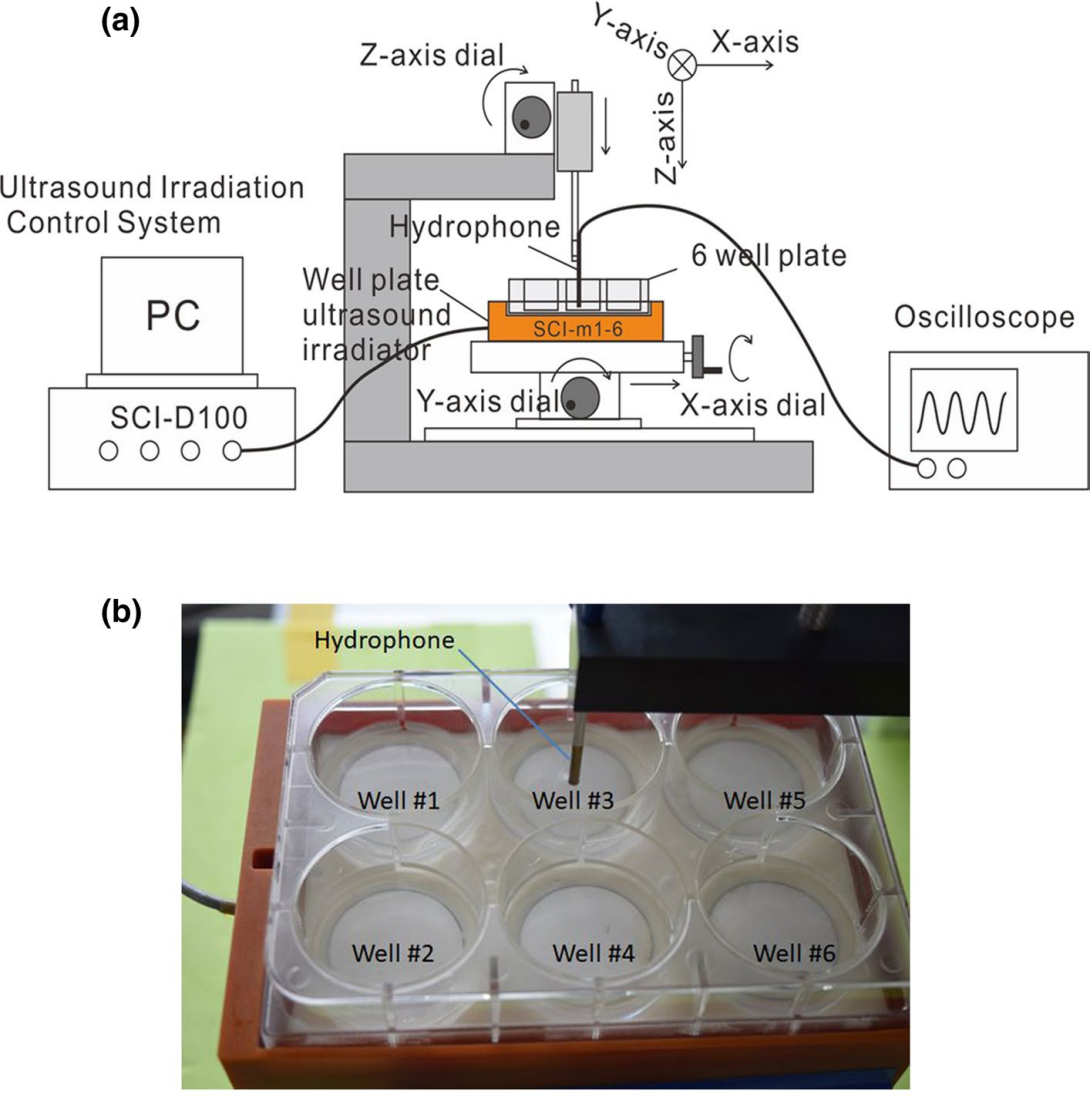
The schema for the method of measurement of ultrasound (US) intensity. The hydrophone was moved in the well filled with degassed water

As it was expected that multiple echoes and standing waves would be generated in this US irradiation system for biofilm, US intensity was measured using the following method. Degassed water was put into a well of a 6-well plate. A hydrophone connected to an oscilloscope was set inside the well of the plate placed on the SCI irradiator. Subsequently, the hydrophone was moved along the well diameter in the *X* and *Y* directions to obtain the distribution of sound pressure in the well. Using the distribution, the spatial-average-temporal-average intensity (*I*_SATA_) was estimated (Fig. 2a, b).

## Examination 1

### Inhibitory effect of long-time US irradiation on biofilm formation

Twenty μL of bacterial culture was added to 2 mL of BHI containing 1% glucose. The culture was then distributed in wells of two 6-well plates. One plate was for US irradiation and the other was for non-US irradiation as a control. US irradiation was performed using the SCI irradiator in an incubator (37 °C and 5% CO_2_). The plate for non-US irradiation was put in the incubator without US irradiation.

US irradiation was of the continuous wave type, and its frequency was 1 MHz. US irradiation time was set at 24 and 12 h. After US irradiation, the amount of biofilm produced on the bottom surfaces of the wells was assessed by crystal violet staining as described earlier.

This examination was repeated twice on other days, and 12 samples were ultimately obtained.

## Examination 2

### Inhibitory effect of short-time US irradiation on biofilm formation

In this examination, the parameters of US irradiation were set according to a previous report that described US irradiation on osteoblasts and showed that irradiation with low-intensity pulsed US enhanced bone fracture healing [12]. In this case, US irradiation parameters were as follows: US intensity, 30 mW/cm^2^; irradiation time, 20 min; US frequency, 1 MHz; and duty ratio, 20%.

**Fig. 3:**
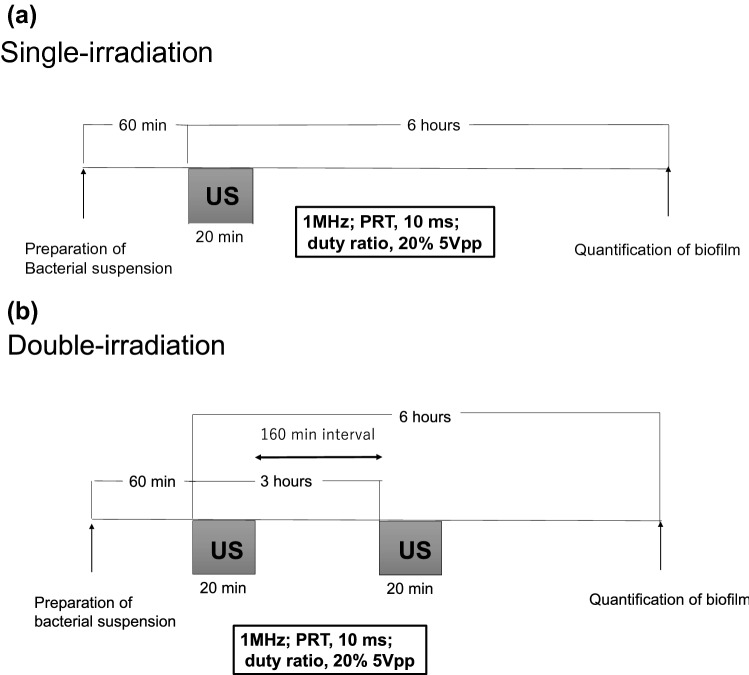
Time course of ultrasound (US) irradiation on the biofilm in “Examination 2” (short-time irradiation). **a** Single irradiation, **b** Double irradiation

Twenty μL of bacterial culture was added to 2 mL of BHI containing 1% glucose. The culture was then distributed in wells of two 6-well plates. The plate for US irradiation was put on the SCI irradiator, and the SCI irradiator along with the plate was put in the incubator set at 37 °C and 5% CO_2_. The plate for non-US irradiation was put in the same incubator at the same time. After one hour, the plate for US irradiation was irradiated with the US for 20 min. US irradiation parameters were as follows: US frequency, 1 MHz; PRT, 10 ms; and duty ratio, 20%. US irradiation was performed once for the single-irradiation group and twice for the double-irradiation group, with a 160-min interval. After this, the plates were further incubated at 37 °C for 6 h for biofilm formation. The amount of biofilm formed was assessed by crystal violet staining as described earlier (Fig. 3a, b). This examination was repeated twice on other days, and 12 samples were obtained.

### Statistical analysis

All statistical analyses were performed using IBM SPSS Statistics for Windows, version 24 (IBM Corp., Armonk, NY, USA). The absorbance of samples at 595 nm is shown as mean ± SD. Statistical significance between the absorbance of the US irradiation group and the non-US irradiation group was determined using a two-tailed unpaired Student’s *t*-test, and values with *p* < 0.01 were considered significant.

## Results

### US intensity in this study

**Fig. 4:**
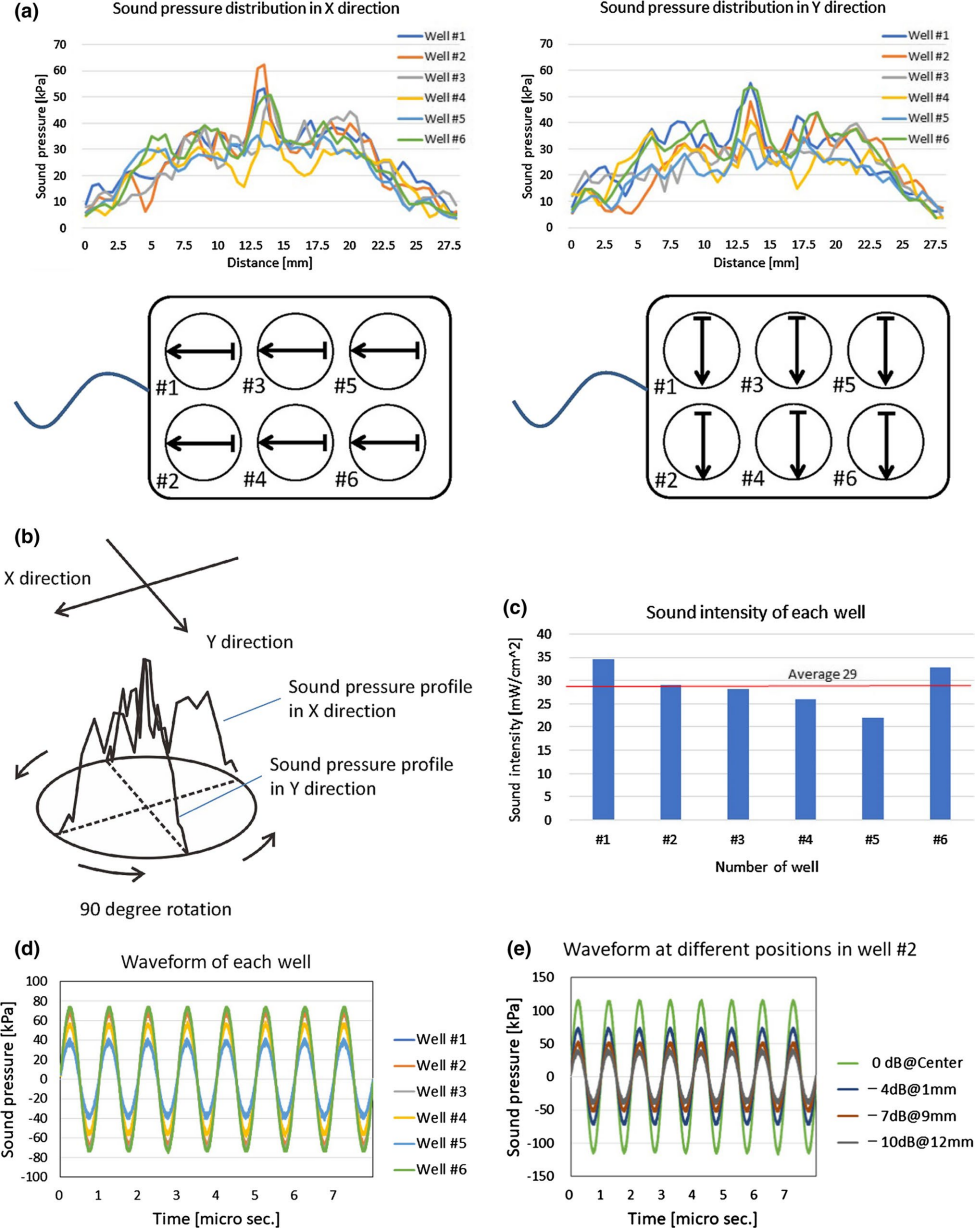
**a** The sound pressure distributions in the *X* and *Y* directions. **b** The total energy of US irradiation in each well was obtained by the rotation manner from the measured sound pressure profiles in both the *X* and *Y* directions as shown in Fig. 4b. *I*_SATA_ was calculated as the total energy divided by the bottom area of the well. **c** Calculated sound intensity of each well. The average in the 6-well plate is 29 $\mathrm{mW}/{\mathrm{cm}}^{2}.$ **d** The sound pressure waveform is observed at the center of each well. The waveforms are substantially sinusoidal. **e** The waveform at different positions (the center of the well and the peripheral positions) in the well. Every waveform even at different positions is also substantially sinusoidal

In “Examination 1”, the US intensity was *I*_SATA_ = 29 mW/cm^2^, whereas, in “Examination 2”, it was *I*_SATA_ = 6 mW/cm^2^ (Fig. 4a–e).

## Examination 1

### US irradiation for 24 h notably reduced biofilm formation

**Fig. 5:**
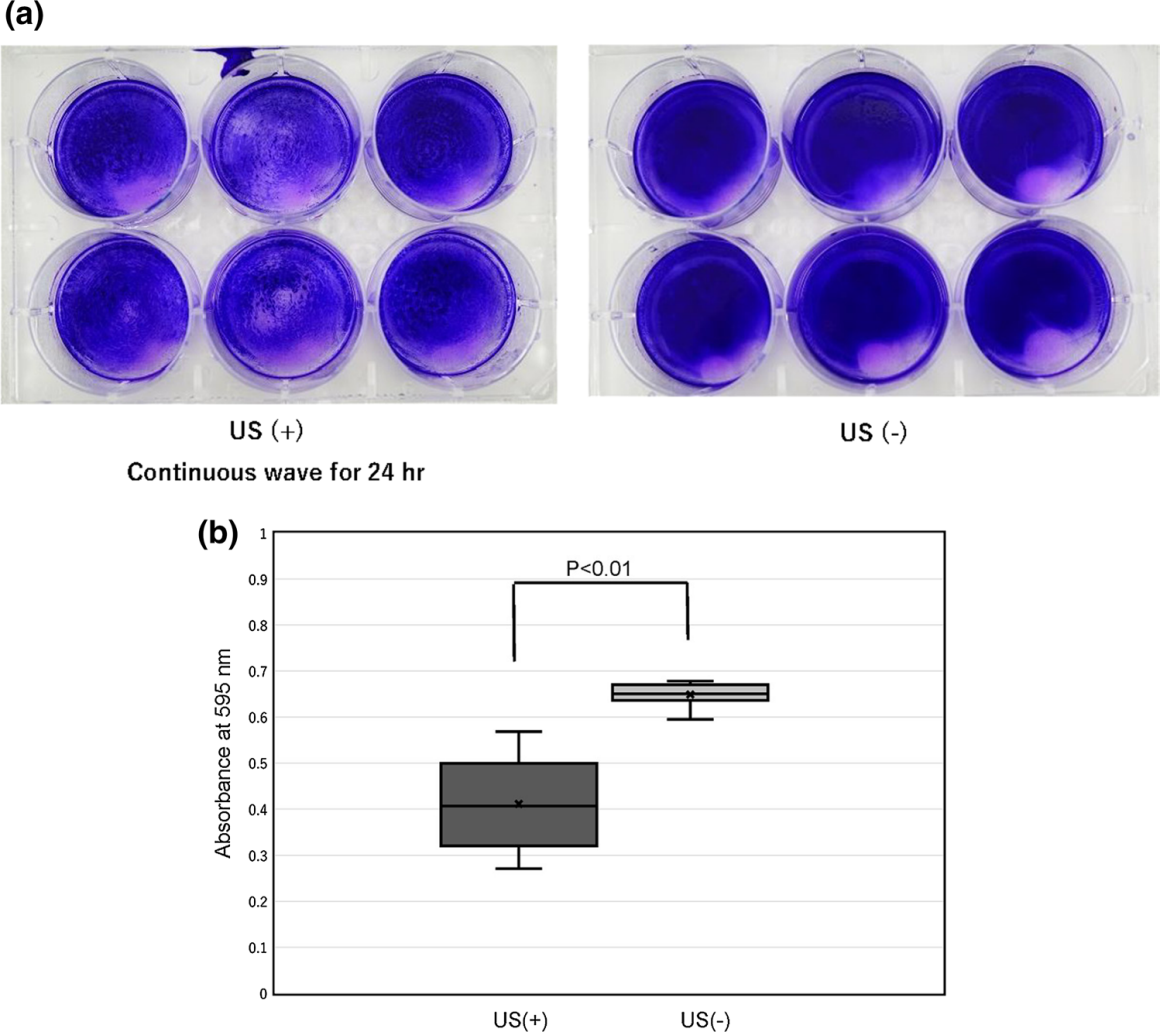
**a** Images of biofilm on the bottoms of the 6-well plates in “Examination 1” (24-h irradiation). Left: US ( +) plate. Right: US (−) plate. The amount of biofilm on the bottoms of US ( +) plates was higher than that of US (−) plates. **b** Comparison of the amount of biofilm formed between the ultrasound (US) irradiation group and the non-US irradiation group in “Examination 1” (24-h irradiation). The amount of biofilm formed was significantly lower in the US irradiation group (US +) than in the non-US irradiation group (US−)

For the samples irradiated for 24 h, the mean absorbance was 0.411 ± 0.099 in the US irradiation group and 0.649 ± 0. 026 in the non-US irradiation group (Fig. 5a, b). The results indicated that the amount of biofilm was significantly reduced because of US irradiation (*p* < 0.01).

**Fig. 6:**
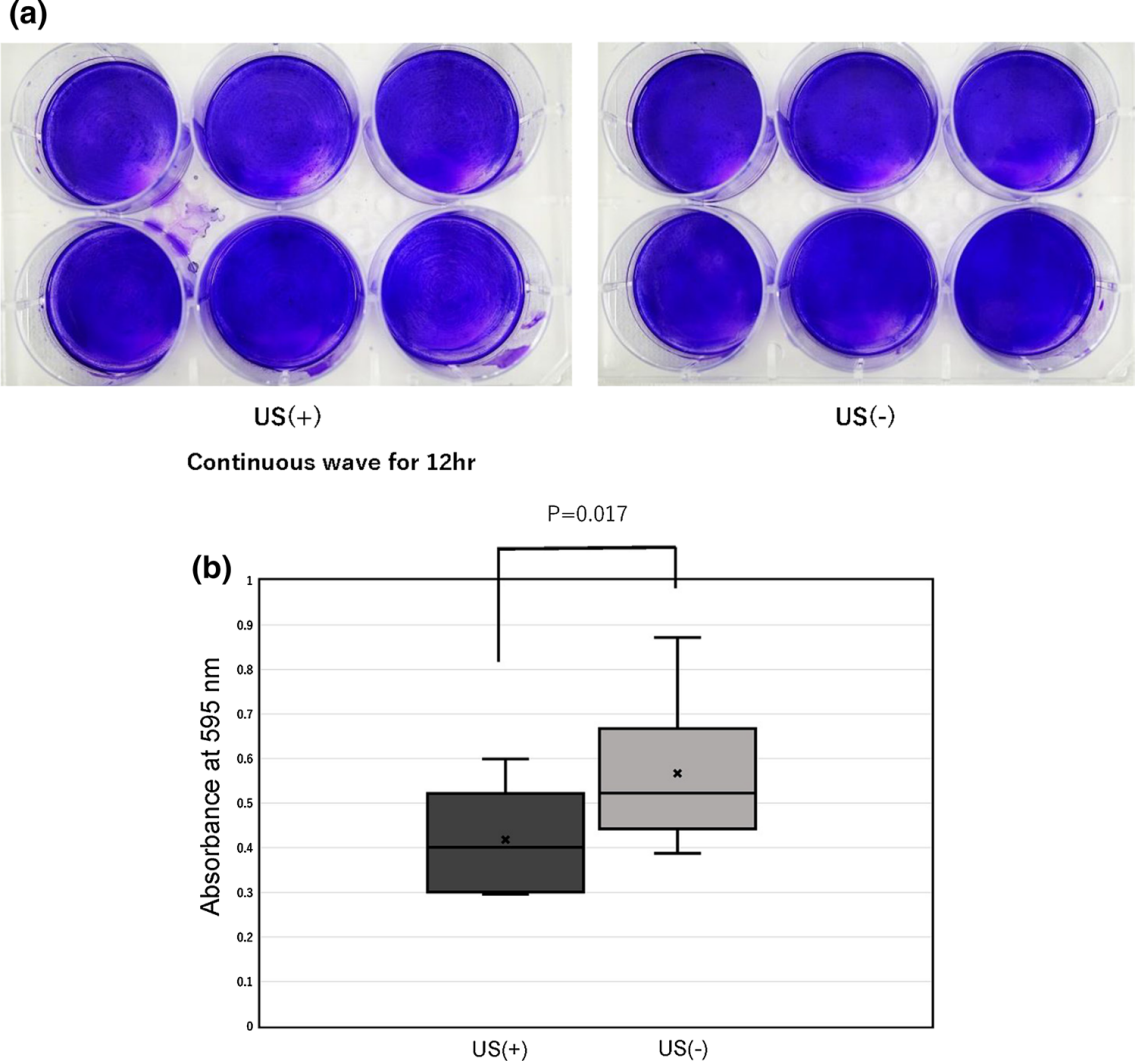
**a** Images of biofilm on the bottoms of the 6-well plates in “Examination 1” (12-h irradiation). Left: US ( +) plate. Right: US(−) plate. There was no difference between the amount of biofilm on the bottoms of US ( +) plates and US (−) plates. **b** Comparison of the amount of biofilm formed between the ultrasound (US) irradiation group (US +) and the non-US irradiation group (US−) in “Examination 1” (12-h irradiation). The change in biofilm formation was not significant between the two groups

For the samples irradiated for 12 h, the mean absorbance was 0.418 ± 0.121 in the US irradiation group and 0.567 ± 0.160 in the non-US irradiation group (Fig. 6a, b). There were no significant differences between the two groups.

## Examination 2

### Short-time single and double irradiation notably reduced biofilm formation

**Fig. 7:**
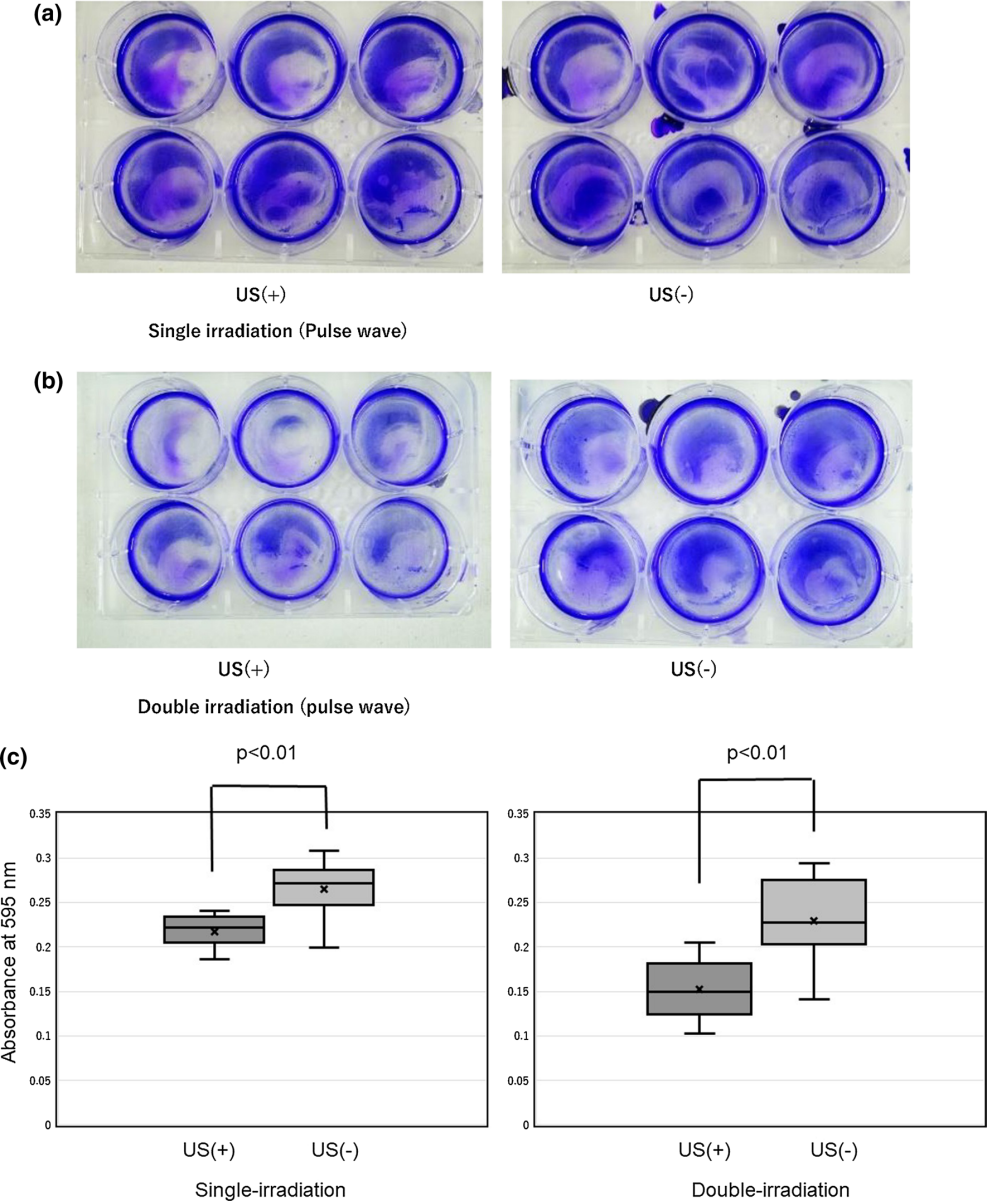
**a**, **b** Images of biofilm on the bottoms of the 6-well plates in “Examination 2”. **a** Single irradiation. **b** Double irradiation. Left: US ( +) plate. Right: US (−) plate. The amount of biofilm on the bottoms of US ( +) plates was higher than that of US (−) plates. **c** Comparison of the amount of biofilm formed between the single and double ultrasound (US) irradiation groups (US +) and the non-US irradiation group (US−) in “Examination 2”. The amount of biofilm formed was significantly lower in the single and double US irradiation groups

In the case of single irradiation, the absorbance of samples was 0.218 ± 0.018 in the US irradiation group and 0.265 ± 0.033 in the non-US irradiation group. The reduction in biofilm formation was 17.9%, and the difference was significant (*p* < 0.01). In the case of double irradiation, the absorbance of samples was 0.153 ± 0.033 in the US irradiation group and 0.230 ± 0.045 in the non-US irradiation group. The reduction in biofilm formation was 33.6%. and the difference, in this case, was also significant (*p* < 0.01). Overall, in both groups, the amount of biofilm decreased notably because of US irradiation (Fig. 7a–c).

## Discussion

The results of this study indicate that US irradiation has inhibitory effects on *S. epidermidis* biofilms.

Previous studies have shown US irradiation influences bacterial biofilms. The US intensity used in these studies was in the order of W/cm^2^; however, this intensity is too high for clinical therapy applications. In this study, US intensities were *I*_SATA_ = 29 mW/cm^2^ and *I*_SATA_ = 6 mW/cm^2^ in Examination 1 and 2, respectively. This intensity is lower than the safe US exposure limit recommended by the FDA.

In addition, US irradiation was used for eradicating biofilm in previous studies, as well as in our study [9, 11]. However, when a biofilm is disrupted, the bacteria present in and around the biofilm could disperse in the bloodstream, resulting in CRBS aggravation. In this study, it was demonstrated that adequate US irradiation could inhibit the formation of biofilm to some extent.

In “Examination 1”, US irradiation for 24 h inhibited biofilm formation, but the same treatment for 12 h did not. This suggests that there is an optimal duration of US irradiation for inhibiting biofilm formation. An application time of 24 h would be inappropriate for hospitalized patients as the extended irradiation exposure may have adverse effects. Therefore, evaluating a shorter optimal duration for irradiation is important.

*S. epidermidis* biofilm formation occurs in two stages: rapid initial attachment of the bacteria to polymer surfaces and cell proliferation combined with the production of polysaccharide intercellular adhesion [5]. In “Examination 2”, US irradiation 1 h after the preparation of bacterial suspension had an inhibitory effect on biofilm formation. This result indicates that US irradiation has an effect on the early stage of biofilm formation, wherein bacteria attach to the bottom surface of the well. Our results concur with those of Wang H et al. [13], who stated that the key factor in stopping biofilm formation was to prevent bacteria from depositing on the medical device surface using ultrasonic vibration. In our study, US irradiation likely produced vibrations on the bottom surface of the well, and this vibration prevented the physical deposition of bacteria on the surface.

In the other studies [14, 15], a greater inhibitory effect was observed from US irradiation combined with antibiotics or microbubbles than that from US irradiation only. During US irradiation, microbubbles are formed, which may act on biofilms and increase their permeability to antimicrobial agents or even kill bacteria in biofilms [14, 15]. If US irradiation can be combined with antibiotics in clinical settings, US intensity can be reduced. In addition, the dose of antibiotics can be reduced, which is particularly beneficial for patients with chronic renal impairment or similar conditions who have a limitation on the dose of medications that can be administered.

There are two limitations to this study. First, the US frequency used in this study was only 1 MHz because of the technical features of the SCI irradiator. If preventing bacteria from depositing on the surface by US vibration is key for the inhibition of biofilm formation, various US frequencies should be evaluated for their effect on biofilm formation. Second, the biofilm used in this study was grown in wells, and not in a flow system. The goal of this study was to apply US irradiation to catheter-inserted vessels for the treatment of CRBSI. Hence, further studies must be conducted on the use of US irradiation on biofilms grown in the bloodstream. It is difficult to experimentally grow biofilm in the bloodstream; therefore, the development of in vivo systems for the formation of biofilm in the bloodstream may be necessary for adaption for therapy in a clinical setting.

In clinical settings, the necessary output power of the US generator will be expectedly higher than that in this study for the following two unavoidable reasons. First, the US is irradiated from the transducer to the catheter from the surface of the skin. In this case, the US wave on the catheter is the traveling wave, not the multiple echoes or the standing wave that were generated in this study. Second, the acoustically effective area on the catheter is smaller than that of the bottom of the well, because the cross-section of the catheter is round, while the bottom of the well is flat.

However, the effective sound intensity discovered in this study is sufficiently small to ensure the safety of living tissue. Hence, the possibility of clinical application can be fully expected. Future work will focus on investigating the most effective conditions for US irradiation and develop a practical method, such as irradiation of catheters, to solve clinical problems in patients suffering from CRBSI.

## Conclusion

Continuous and pulsed wave US irradiation show potential for the inhibition of *S. epidermidis* biofilm formation at a level below the FDA’s recommended therapeutic US intensity level. Continuous-wave US irradiation significantly inhibited biofilm formation after 24 h of exposure, but not after 12 h, while pulsed-wave US irradiation applied in two 20-min intervals produced a significant 33.6% reduction in biofilm formation. US irradiation could be a novel method for preventing bacteria from forming biofilms, and this could be useful in treating CRBSIs caused by bacteria in biofilms.
